# mPalliative Care Link: Examination of a Mobile Solution to Palliative Care Coordination Among Tanzanian Patients With Cancer

**DOI:** 10.1200/GO.21.00122

**Published:** 2021-08-18

**Authors:** Mamsau Ngoma, Beatrice Mushi, Robert S. Morse, Twalib Ngoma, Habiba Mahuna, Kaley Lambden, Erin Quinn, Sarah B. Sagan, Yun Xian Ho, F. Lee Lucas, Joshua Mmari, Susan Miesfeldt

**Affiliations:** ^1^ Ocean Road Cancer Institute, Dar es Salaam, Tanzania; ^2^ Muhimbili University of Health and Allied Sciences, Dar es Salaam, Tanzania; ^3^ Da Vinci Usability Incorporated, Lexington, MA; ^4^ Dimagi Incorporated, Cambridge, MA; ^5^ Maine Medical Center, Portland, ME

## Abstract

Late-stage cancer patient symptom control is a national priority in Tanzania. Mobile health promises to improve the reach of a limited pool of palliative care specialists through interprofessional, community-based care coordination. This work assessed the effectiveness of a smartphone- or Web-based app, mPalliative Care Link (mPCL), to extend specialist access via shared data and communication with local health workers. Central to mPCL is the African Palliative care Outcome Scale (POS), adapted for automated mobile symptom assessment and response.

**METHODS:**

Adult patients with incurable cancer were randomly assigned at hospital discharge to mPCL versus phone-contact POS collection. Sociodemographic, clinical, and POS data were obtained at baseline. Twice-weekly POS responses were collected and managed via mPCL or phone contact with clinician study personnel for up to 4 months, on the basis of study arm assignment. Patient end-of-study care satisfaction was assessed via phone survey.

**RESULTS:**

Forty-nine patients per arm participated. Comparison of baseline characteristics showed an insignificant trend toward more women (*P* = .07) and higher discharge morphine use (*P* = .09) in the mPCL group compared with phone-contact and significant between-group differences in cancer types (*P* = .003). Proportions of deaths were near equal between groups (mPCL: 27%; phone-contact: 29%). Overall symptom severity was significantly lower in the phone-contact group (*P <* .0001), and symptom severity decreased over time in both groups (*P* = .0001); however, between-group change in overall symptoms over time did not vary significantly (*P* = .34). Care satisfaction was generally high in both groups.

**CONCLUSION:**

Higher symptom severity scores in the mPCL arm likely reflect between-group sociodemographic and clinical differences and clinical support of phone-contact arm participants. Similar rates of care satisfaction in both groups suggest that mPCL may support symptom-focused care coordination in a more efficient and scalable manner than phone contact. A broader study of mPCL's cost efficiency and utility in Tanzania is needed.

## INTRODUCTION

In sub-Saharan Africa, there are at least 500,000 cancer deaths/year with a projected doubling of mortality rates by 2030.^1-6^ A two-country study showed unnecessary suffering among African patients with late-stage cancer, often uncontrolled pain.^7^ Relevant to countries such as Tanzania with high cancer mortality rates,^8^ palliative care (PC) improves patient and caregiver outcomes, including patient quality of life (QoL) and caregiver burden.^9^ Furthermore, studies in the high-resource setting reveal prolonged life expectancy among patients with cancer offered specialized PC.^10^ PC access for patients with cancer is a Tanzanian priority, calling for evidence-based, culturally relevant, and scalable community-based solutions because of a limited pool of PC specialists and restricted access to support resources.^11-13^ As 66% of Tanzania's population is rural,^14^ research must be focused on the geographically remote.

**CONTEXT**

**Key Objective**
Can mobile-health improve the reach of a limited pool of palliative care (PC) specialists treating patients with cancer through remote care coordination with local health workers in resource-constrained settings?
The smartphone-deployable mPalliative Care Link (mPCL) app was designed for automated symptom-focused assessment and response-based care coordination and tested versus phone-contact symptom assessment in an urban Tanzanian setting.
**Knowledge Generated**
Measures of physical and emotional symptoms were higher (reflecting higher symptom burden) in the mPCL arm, likely reflecting the lack of a true usual-care arm and between-group clinical and sociodemographic differences; however, patient satisfaction with the care provided was high in both arms.
**Relevance**
A broader study of mPCL's cost -efficiency and utility is needed in Tanzania. This work holds promise for closing a large PC gap among patients with cancer in underresourced settings globally and serves as important baseline data as PC needs evolve throughout and beyond the COVID-19 pandemic.

With increasing adoption of mobile-health (mHealth) technology in Tanzania, there is potential to improve cancer symptom control and QoL through remote PC access by facilitating interprofessional communication and care coordination with community-based local health workers (LHWs) or other generalists.^15^ In close partnership with representatives of three user groups (ie, Tanzanian patients with cancer and their lay caregivers [hereafter, caregivers]; PC specialists [hereafter, specialists]; and LHWs), we developed a smartphone-deployable app prototype, mPalliative Care Link (mPCL), extending the reach of specialists through remote symptom-focused assessment and response-based care coordination. Full description of mPCL prototype development is reported elsewhere.^16^ Here, we report outcomes of an mPCL field test among patients from a single, urban, government-supported hospital, Ocean Road Cancer Institute (ORCI).

## METHODS

The Muhimbili University of Health and Allied Sciences Institutional Review Board approved this work. Informed consent was required and obtained from participants before study enrollment.

### Description of the mPCL Intervention

mPCL was built on an open-source, secure cloud-based platform, CommCare,^17^ developed to be accessed through a native mobile app on an Android device or as a Web app on a desktop computing device. mPCL functionalities are summarized in Table 1.

**TABLE 1:**
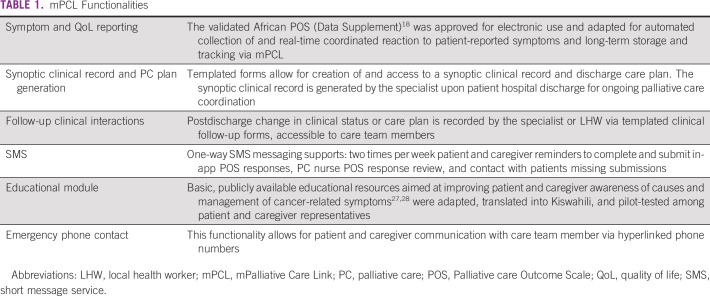
mPCL Functionalities

### Setting

ORCI, the largest government-supported cancer center in Tanzania, treated 6,161 new and 48,546 follow-up patients in 2019 (author M. Ngoma, personal communication, January 2021). Its Palliative Care Service offers inpatient and outpatient services, including select pain medications (ie, morphine and tramadol) free of charge.

### Participants

Participants included six ORCI-based specialists (five oncologists and one nurse), 10 LHWs with experience caring for ORCI patients, and 98 patients with cancer and their caregivers.

Specialists and LHWs were identified, contacted, and consented by clinician study team members. Patients were eligible for the field test if they were ORCI adult inpatients with untreatable malignancies (regardless of stage), due for discharge. They were required to have at least a 4-month life expectancy per specialist assessment, as well as a caregiver and LHW consenting to support outpatient management for field-test duration, experience with mobile devices (measured by cell phone ownership), and completed primary school for study material comprehension and to reside within 50 km of ORCI for medication and clinical support access.

ORCI specialists identified potential field-test patients before hospital discharge and provided names to clinician study team members to determine study eligibility and interest. Patients consenting to study were randomly assigned to one of two field-test arms in a 1:1 ratio using a computer-generated random assignment, transferred to sequentially numbered opaque envelopes. The field-test arms were *mPCL arm*, where Palliative care Outcome Scale (POS) response collection was automated two times per week, or *phone-contact arm*, where POS responses were collected by a clinician study team member via cell phone two times per week.

**FIG 1:**
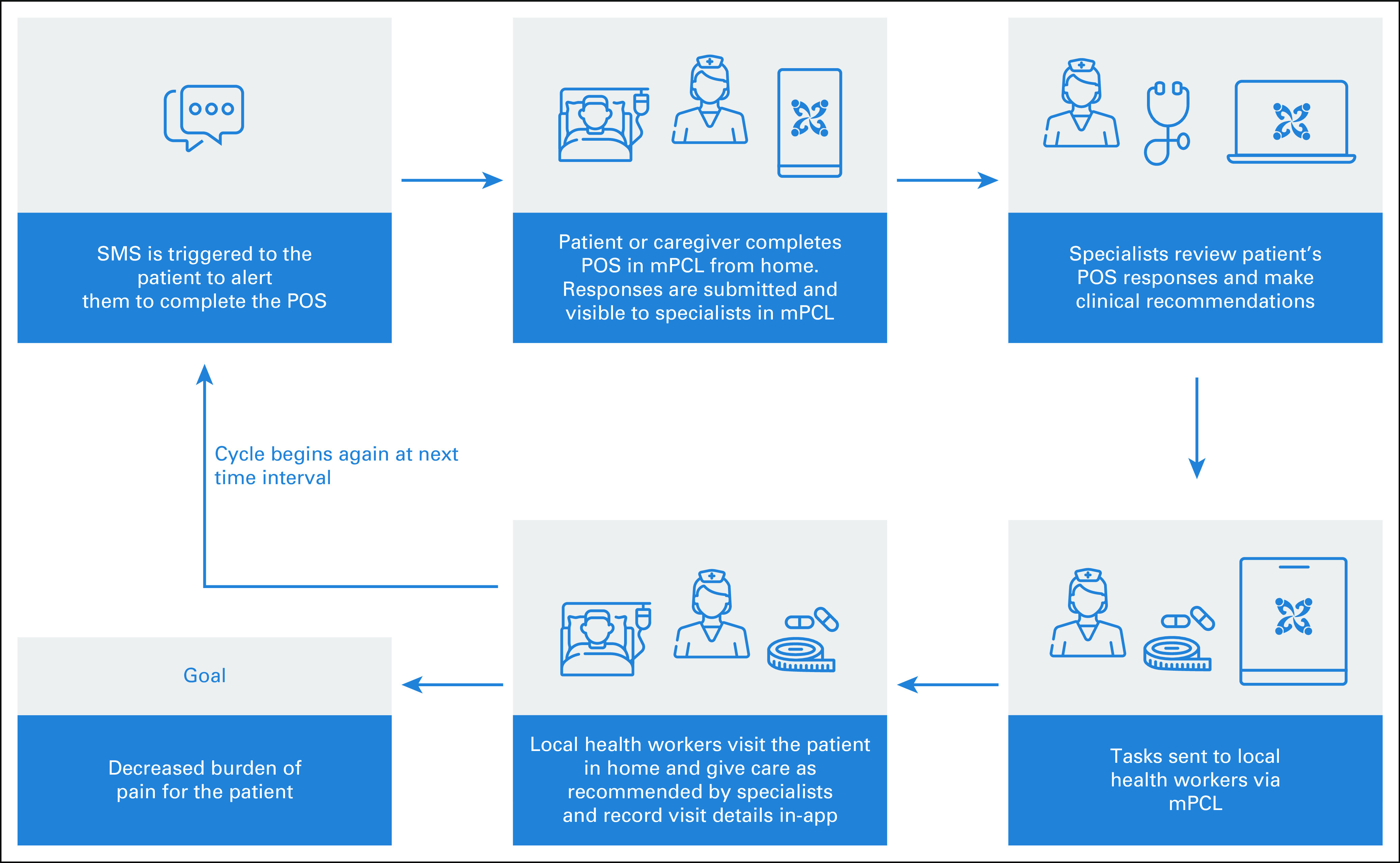
mPCL-directed care coordination. mPCL, mPalliative Care Link; POS, Palliative care Outcome Scale; SMS, short message service.

An Android device was configured for each mPCL arm patient by the study IT coordinator. All patients and caregivers received in-person training on how to use mPCL to (1) complete and submit POS responses, (2) anticipate mobile communication from the specialist or LHW in response to worrisome POS results or (3) learn about planned in-person assessments by the LHW or PC nurse in response to escalating symptoms (Fig 1). Phone-contact arm patients were informed that they would receive calls two times per week from a clinician study team member for POS response collection. All patients could access usual-care ORCI PC services. Government-supported LHWs are available throughout the greater Dar es Salaam region. mPCL arm patients were assigned an LHW, whereas phone-contact arm patients received usual-care LHW support. Participation lasted up to four months.

### Technology

mPCL was installed on LHWs' and patients' personal devices, and those lacking a suitable device were loaned an Android smartphone with mPCL for the study duration. Specialists accessed mPCL on an office-based, secure PC, a tablet, or their personal Android smartphones.

### Survey Instruments

#### POS.

**FIG 2:**
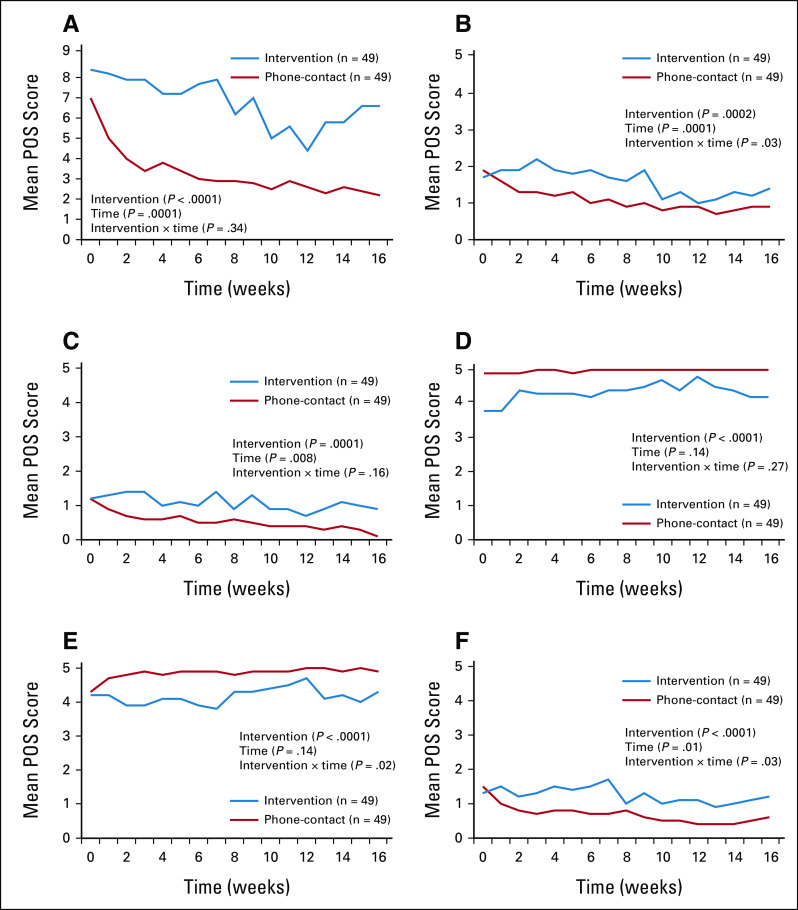
QoL comparisons: intervention (mPCL) versus phone-contact groups. POS scores. (A) Overall (total) POS scores scaled from 0 to 35 (low to high symptoms) averaged across patients randomly assigned to either the intervention (mPCL use) or phone-contact group are shown at the top left over time, with individual POS assessment items (B-F) pain, worry, feels life is worthwhile, feels at peace, and other symptoms scaled from 0 to 5 (low to high symptoms) shown separately. Significance levels for intervention, time, and intervention × time interactions are shown for each item. mPCL, mPalliative Care Link; POS, Palliative care Outcome Scale; QoL, quality of life.

The previously validated African POS includes 10 Likert-scaled (ie, from 0 to 5), Kiswahili-language items: seven patient-focused and three caregiver-focused. We report on patient-focused items (Data Supplement).^18^ The two physical symptom–based and worry-based items (ie, items 1-3) are coded such that *higher scores mean poorer outcomes*. Remaining items are coded such that *higher scores mean better outcomes* (ie, items 4-7). For the purpose of reporting the overall scores for both groups (Fig 2), we reverse-coded items 4-7 for this one composite measure (maximum = 35), ie, a higher measure on the total POS score is worse.

#### End-of-study, quality-of-care survey.

Patients or caregivers as proxy completed a phone-delivered 11-item survey (10 multiple choice and one open response) focused on perceived field-test quality of care. mPCL arm respondents and caregivers reflected on care provided via mPCL, whereas phone-contact patients and caregivers reflected on care provided by their health worker (including traditional healers). This survey was adapted from the VA PROMISE Center instrument,^19^ including (1) adjustment of questions to reflect study setting; (2) ORCI-based team members' input regarding language, content, and cultural relevance; (3) development of two versions reflecting study arms; (4) Kiswahili translation with back translation and accuracy review; and (5) usability testing among seven ORCI patients with cancer.

The end-of-study, Likert-scaled items were coded such that higher *scores mean poorer perceived care quality*. For consistency of reporting, items were reverse-coded such that *higher scores mean high quality of care.*

### Data Collection

All data were collected, coded, and stored securely via CommCare (ie, mPCL system usage and POS surveys) and a password-protected computer at ORCI (ie, end-of-study survey); de-identified data sets were used for analysis.

#### Sociodemographic data.

Sociodemographic data were collected from patients as were basic sociodemographic and practice characteristics from specialists and LHWs (clinician data not reported).

#### Clinical data.

Among patient participants, select clinical data and baseline POS responses were collected and recorded by a clinician team member. Key clinical data were used to generate the mPCL synoptic clinical record and PC plan upon hospital discharge among mPCL-randomized patients.

#### QoL assessment (POS).

Patients and caregivers assigned to the POS intervention received automated short message service (SMS) reminders to complete and submit the POS via mPCL each Monday and Thursday. Patients and caregivers not responding received a follow-up SMS reminder 24 hours later. Furthermore, automated SMS reminders were sent to the PC nurse to review patient POS submissions every Tuesday and Friday. Lack of response to POS response requests prompted a phone call from a clinician study team member wherein POS responses were collected and entered into mPCL. Caregivers served as proxy for patients unable to complete the POS. POS results were accessible to specialists and LHWs for review, reaction, and ongoing tracking and final field-test analysis.

Phone-contact arm patients were called by a clinician study team member two times per week to collect POS responses with timing corresponding with automated mPCL POS delivery. Patients and caregivers could voice concerns and worries; clinician team member addressed questions and offered support. Report of intolerable pain or other perceived life-threatening symptoms among phone-contact patients prompted contact with ORCI Palliative Care Service staff for follow-up.

#### Medication use.

Postdischarge, medication data were updated in mPCL by the specialist, on the basis of mPCL-collected POS responses, phone or in-app communication with the patient or LHW, or clinic or hospital follow-up. Among patients in the phone-contact group, medication usage data were collected and recorded two times per week during POS response collection.

#### End-of-study survey.

The end-of-study survey was collected by clinician study team members via cell phone from all patient participants or proxy caregivers.

### Data Analysis

Random assignment success was assessed via comparison of baseline characteristics (including baseline POS scores) using *t* tests or the nonparametric equivalent as appropriate for continuous variables and chi-square tests, or Fisher's exact tests for categorical variables. Because of varying numbers of responses/patient, we first took the mean of all POS responses for each patient weekly. To describe changes, we used means and standard deviations to summarize pain and other POS score trajectories for both patient groups. Pain and other POS scores were further analyzed using repeated measures analysis of variance estimating the time effect, the treatment (mPCL *v* phone-contact) effect, and the time × treatment interaction (the effect of interest, testing the hypothesis that POS trajectories over time differ by treatment group). To accommodate different numbers of observations/patient (ie, some patients did not report for some weeks and some patients died before the end of study), we used mixed models to estimate the repeated measures models, with a random effect for the patient to account for within-patient correlation, using an autoregressive correlation structure.

We describe the results of individual POS items, reflecting physical, emotional, and overall symptom (ie, total) scores at baseline and over time as indicators of QoL by group. We examined differences in discharge and follow-up medications. Because medication information was collected differently in the two groups, these results are suggestive. The number of deaths and time to death were tracked, comparing between-group differences.

Likert-scaled responses from the end-of-study survey were summarized using means and standard deviations and compared, between groups, using independent sample *t* tests.

## RESULTS

### Sociodemographic and Disease Characteristics

There were 49 patients/arm (Table 2). Although the two study populations were largely matched, there were some differences, including a nonsignificant trend toward more women versus men in the mPCL arm versus the phone-contact arm (*P* = .07) and significant differences in self-reported profession (*P* = .004) and cancer type (*P* = .003) (ie, more cervical cancer cases in the mPCL arm).

**TABLE 2:**
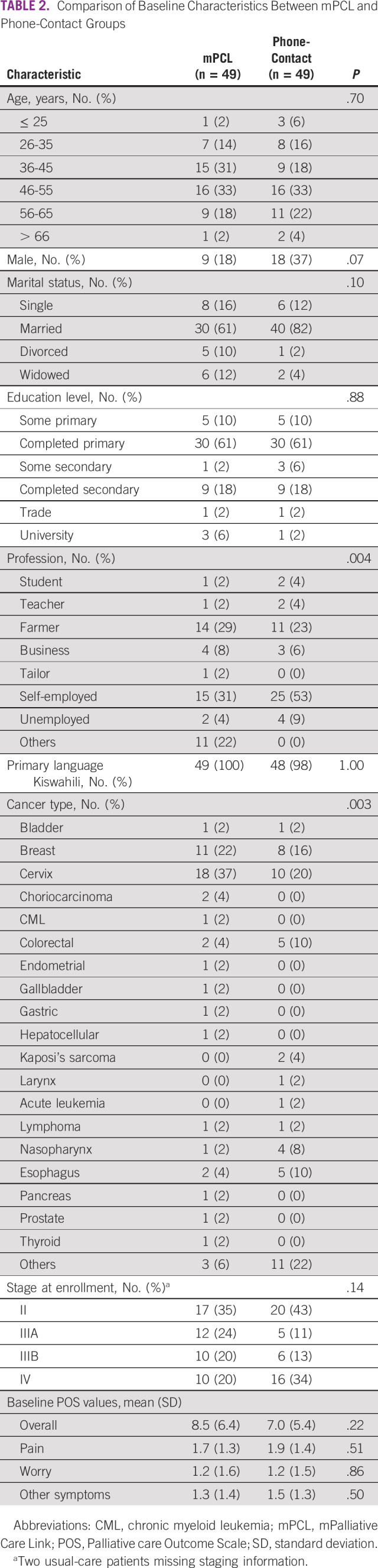
Comparison of Baseline Characteristics Between mPCL and Phone-Contact Groups

### Deaths

There were similar numbers of deaths during the study period, comparing arms (ie, 14 deaths [29%] in the phone-contact arm *v* 13 deaths [27%] in the mPCL arm [*P* = 1.00]). The median number of days to death was 45 (interquartile range: 26-66) in the phone-contact arm versus 61 (43-86) in the mPCL group.

### POS Responses

The average weekly number of POS responses were similar, ie, 1.75 and two in the mPCL and phone-contact arms, respectively, with similar ranges (minimum 1.15 in mPCL *v* 1.0 in phone-contact; maximum 2.26 in mPCL *v* 2.33 in phone-contact).

### POS Physical Symptoms Findings

Figure 2 illustrates that the overall POS summary score (ie, total POS score) for the patient-directed items were lower among those in the phone-contact arm versus those in the mPCL arm (*P* < .0001), *indicating significantly higher QoL* in the phone-contact arm. Individual measures of pain and other symptoms revealed significantly higher scores (ie, more severe symptoms) in the mPCL arm versus the phone-contact arm (*P* = .0002 and *P* < .0001 for pain and other symptoms, respectively). Physical symptoms improved throughout the 16-week study period, as reflected in a decrease of indicator scores in both populations with no between-group differences over time in total POS scores but slightly greater improvement in the phone-contact arm for pain and other symptoms.

### POS Emotional Symptoms Findings

Shown in Figure 2, phone-contact arm patients had significantly lower worry scores (*P* = .0001), greater feelings of peace (*P* < .0001), and a greater sense that life seemed worthwhile than mPCL patients (*P* < .0001); worry scores improved to an equal extent over time comparing groups. All patients in both study populations reported high levels of feeling at peace and that life is worthwhile throughout the study period, with extreme ceiling effects found (Fig 2).

### Medication Use

Comparing discharge medications between the two populations, a greater percentage of mPCL arm patients received discharge morphine versus the non-mPCL group (22% *v* 8%, respectively); however, this difference did not meet statistical significance (*P* = .09; data not shown). In contrast, the phone-contact group was more likely to receive tramadol at discharge versus the mPCL group (31% *v* 2%, respectively, *P* = .0004; data not shown). As the method of collecting medication data postdischarge varied, it was difficult to compare groups; however, equal numbers (31% in each arm; *P* = 1.00) of patients in each group reported using morphine during the study period.

### Quality of Care

Table 3 summarizes the nine Likert-scaled responses to the end-of-study care satisfaction survey. The tenth (yes or no response) survey item, not shown in Table 3, asked if the patients experienced pain or required pain medications. With the exception of one mPCL arm participant, all respondents replied yes. Table 3 shows overall satisfaction with the care delivered in both groups. Differences included significantly higher satisfaction with staff response to questions and concerns in the phone-contact group and significantly more spiritual support in the mPCL group.

**TABLE 3:**
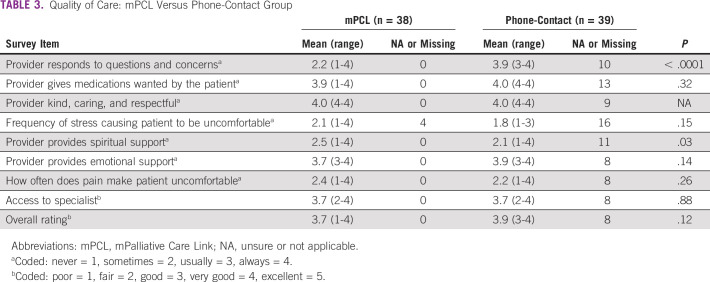
Quality of Care: mPCL Versus Phone-Contact Group

## DISCUSSION

The role of mHealth to support cancer care delivery has gained increasing attention over the years with the advancement of digital technology allowing for the development of innovative solutions to address gaps in the cancer care continuum.^20^ Although there is growing literature backing mHealth use in oncology in the high-resource setting, there is a dearth of evidence on mHealth utility in addressing the rising burden of cancer in low- and middle-income countries.^21-23^ As a majority of cancer is diagnosed at an advanced stage, there is a focus on end-of-life PC in the low-resource setting.^22^ In Tanzania, despite increased efforts to deliver PC services to patients with cancer, access to specialists and resources for pain control, and attention to other physical and emotional symptoms continue to be severely limited or nonexistent,^24,25^ calling for creative solutions (ie, mHealth).

Although previous studies have examined mHealth impact on cancer-related QoL, to our knowledge, this is the first study of a mobile app developed and tested within a low-resource setting in sub-Saharan Africa. In an earlier systematic review of mHealth literature focused on the QoL support of patients with cancer in the high-resource setting, of the 4,929 articles retrieved, 20 RCTs were examined in the final review. These studies were heterogeneous in intervention type, duration, and outcome measures. The focus of these studies varied including examination of the effect of telehealth on physical and emotional QoL at different points in the cancer care trajectory. Two of the three studies focused on pain control revealed significant benefit of the telehealth intervention, four of the nine studies dedicated to depression showed significant intervention benefit, and studies focused on overall QoL demonstrated significant effects. Importantly, the majority of the mHealth interventions included were telephone-based.^26^ Although the cost of cell phones has decreased, the limited availability and expense of maintaining a professional workforce to deliver phone-based care is a major barrier to this form of PC telehealth support in Tanzania and strengthens the need for innovative solutions such as mPCL. Other challenges to mHealth, particularly in the rural setting, include lack of electricity and unreliable Internet coverage.

Here, we describe the outcomes of an mPCL field test focusing on care coordination between a finite number of specialists located at a large, urban, government-supported cancer center and a cohort of community-based LHWs. We compared QoL (physical and emotional) outcomes in two study arms and found significantly higher POS scores (ie, greater symptom burden) in the mPCL arm. Although this is unexpected given the intent of the app to support symptom management, study design and review of field-test data reveal likely reasons for findings.

First, because of ethical and feasibility concerns with the assignment of a true usual-care arm, ie, POS responses collected without clinical oversight or support, mPCL intervention outcomes were compared with those in the phone-contact arm where POS responses were collected via direct phone communication with a clinician study team member, with timing corresponding with automated app-based POS collection for direct comparison. This is important to acknowledge as mPCL QoL outcomes were measured against human phone contact and support twice-weekly, which likely served as its own intervention above and beyond usual care. In contrast, direct contact with the mPCL participants only occurred at the time of escalating symptoms. As a result, control patients may have indeed felt more cared for, reflected in better POS scores. As mHealth interventions are expected to grow and to increasingly use artificial intelligence and machine learning in the support of the individual patient, it is essential to include an assessment of patients' comfort with this evolving technology in comparison with direct human-to-human contact, particularly in the provision of high-quality PC.

Second, although study arm assignments were randomized, Table 2 shows several important between-group sociodemographic and clinical differences. Specifically, more women and greater numbers of cervical cancer cases were assigned to the mPCL arm and reported profession also varied. Furthermore, there was greater use of discharge morphine among mPCL patients, potentially indicating higher baseline symptoms or extent of disease. Despite these differences, groups did not vary in interval deaths within the study period. Although these sociodemographic and clinical differences are likely because of the small sample size and divergent cancer types, it is possible that they explain a higher symptom burden in mPCL patients.

Unexpectedly, despite limited treatment options, we found a significant decrease in physical symptom levels over the study period in both groups corresponding to a gradual *increase* in QoL. This gradual improvement likely reflected cumulative deaths of those with the highest symptom burden.

Furthermore, the decline in symptoms may have reflected the symptom-directed clinical support offered to both study populations and their caregivers. Similar findings were reported in a study assessing the impact of home-based PC among geographically diverse Tanzanian patients with cancer.^24^

Our data reflecting worry, not feeling at peace, and the sense that life is not worthwhile in both groups mirror those of overall symptom burden, with greater emotional symptom burden in the mPCL versus phone-contact group. Similar to physical symptoms, there was a steady decline in worry over time and maintenance of between-group worry differences. This reduction in worry may have partially reflected the cumulative death of patients; however, this decline occurred early. Thus, it may have reflected the patient's discharge from hospital to home where they were surrounded by loved ones. POS-reported sense of peace and the feeling that life is worthwhile were high in both groups and stayed high throughout the study period, likely reflecting ceiling effects. The emotional status of both arms may have reflected access to those within the home and the clinical support offered to both arms.

As between-group sociodemographic and clinical differences and the lack of a true usual-care arm make it difficult to come to conclusions regarding the impact of mPCL on QoL, responses to the end-of-study survey instrument provide valuable insight into the potential benefit of mPCL in supporting the PC needs of Tanzanian patients with cancer.

Specifically, there was generally high care satisfaction in both groups, with minor differences in comparing responses. Most highly ranked in both were the following: staff was kind, caring, and respectful; medications were made available; specialist was accessible; and emotional health was supported. Although we do not have baseline care satisfaction data and lacked a true usual-care group, overall high levels of satisfaction in both study arms indicate need for further mPCL study.

It is noteworthy that across all POS-measured indicators, symptoms were lower than expected in both study arms, contrasting with published data revealing significant symptomatology among sub-Saharan African patients with cancer.^7^ Specifically, the overall score remained lower than 9 (maximum of 35) throughout the study period in both arms. Regarding pain, with the exception of week 3, average pain scores in both study populations fell below 2 (maximum = 5), and other symptom scores were below 1.8 (maximum = 5) throughout the study period. Given the clinical status of the patients enrolled, all emotional symptoms were also lower than expected. It is possible that the physical and emotional symptoms measured reflected the study-based care provided to both populations. Furthermore, lower than expected symptomatology may reflect the availability of existing ORCI Palliative Care Service resources or a combination of study-delivered support coupled with institutional resources. POS-reported scores may have also reflected patients' and caregivers' desire to please their clinical caregivers and study personnel. Sparse baseline ORCI and Tanzanian QoL data limit the investigators' ability to determine factors reflecting symptom scores reported and reveal the need for a broader study of mPCL. Other study limitations include small sample size, patients from a single urban cancer hospital, and the lack of a true usual-care group.

Careful assessment of the cost of mHealth PC solutions is essential to the future utility and scalability of resources such as mPCL. Furthermore, although the work described here was completed in advance of the COVID-19 pandemic, our results should serve as an important baseline against which to compare future acceptance of virtual PC in the setting of ongoing threats to in-person visits posed by COVID-19 and other pathogens.

We report early field-test experience with a smartphone- or Web-based app extending specialist access via shared care with community-based LHWs, aimed at improved end-of-life cancer symptom and QoL control. To our knowledge, this is the first mobile app focused on end-of-life symptom-based care coordination within sub-Saharan Africa.

Higher symptom scores in the mPCL intervention arm are likely due, in part, to the lack of a true usual-care arm combined with notable between-group sociodemographic and clinical differences. Near-equal care satisfaction between the two groups suggests the app's potential value to provide comparable care to clinician-led phone support in a more resource-efficient and scalable manner. A larger randomized study of the app is needed to further investigate its clinical utility and cost-effectiveness, particularly in the rural setting with fewer referral systems in place and limited access to medications. This work holds promise for closing a large PC gap in underresourced settings and serves as important baseline data as PC needs evolve throughout and beyond the COVID-19 pandemic.
